# Application of the Unified Protocol for Transdiagnostic Treatment of Emotional Disorders in a Group Format for Adults: A Scoping Review Protocol

**DOI:** 10.3390/bs15030389

**Published:** 2025-03-19

**Authors:** Machiko Kajiwara, Noriko Kato, Motohiro Nishiuchi, Hiroko Fujisato, Kyousuke Kaneko, Hironori Kuga, Masaya Ito

**Affiliations:** 1 National Center for Cognitive Behavior Therapy and Research, National Center of Neurology and Psychiatry, Ogawa Higashi 4-1-1, Kodaira 187-8511, Tokyo, Japan; machikokajiwara@ncnp.go.jp (M.K.); nishiuchi.m@ncnp.go.jp (M.N.); kyosukekaneko@ncnp.go.jp (K.K.); hirokuga@ncnp.go.jp (H.K.); masayait@ncnp.go.jp (M.I.); 2 Faculty of Sociology, Kansai University, Yamate 3-3-35, Suita 564-8680, Osaka, Japan; fujisato@kansai-u.ac.jp

**Keywords:** cognitive behavioral therapy, transdiagnostic approach, Unified Protocol, group psychotherapy, emotional disorders

## Abstract

The group format of the Unified Protocol for Transdiagnostic Treatment of Emotional Disorders (UP) shows promise as a efficient method for delivering established and effective psychological treatments for emotional disorders. The implementation of psychological treatment in a group format varies according to a country’s local circumstances, policies, and culture. To date, there are no comprehensive reviews of aspects related to settings, participants, providers, and interventions for applying the UP in a group context. Therefore, we have prepared a scoping review protocol to clarify the fields, participant characteristics, intervention structures, and therapist training methods related to group-format UP, aiming to facilitate its implementation across diverse regions and contexts. Based on this protocol, the scoping review will follow the Joanna Institute guidelines and PRISMA statement extensions for scoping reviews. The review will include studies of the group format of UP that describe symptoms of emotional disorders in adults. The following databases will be searched: PubMed, MEDLINE, Web of Science, PsycINFO, and ClinicalTrials.gov. The selected data will be organized according to four predefined research questions. This scoping review will provide suggestions to promote the application and implementation of the UP in a group format and contribute to disseminating evidence-based psychological treatment.

## 1. Introduction

Depressive and anxiety disorders are common mental disorders with high prevalence (COVID-19 Mental Disorders Collaborators, 2021). These disorders not only cause substantial psychological and physical distress but also lead to social isolation and socioeconomic loss due to increased healthcare costs and decreased productivity (König et al., 2019; Konnopka & König, 2020). The Global Burden of Disease study reported that these disorders are mental disorders that cause the largest disease burden (COVID-19 Mental Disorders Collaborators, 2021). It was also reported that the burden of depressive and anxiety disorders overall increased from 19th and 34th place in 1990 to 13th and 24th place in 2019, increases of 61.1% and 53.7%, respectively (GBD 2019 Diseases and Injuries Collaborators, 2020).

Cognitive behavioral therapy (CBT) is an effective psychological treatment for depressive and anxiety disorders (Carpenter et al., 2018; Cuijpers et al., 2020). CBT has the potential to reduce the disease burden of these disorders on individuals and society (Santomauro et al., 2023; Vos et al., 2004), although there are obstacles to its dissemination and implementation. Traditionally, CBT protocols are designed to be disorder-specific, with many of the manuals developed to target specific conditions based on established diagnostic classifications (Hofmann et al., 2012). Although there is strong evidence supporting the effectiveness of disorder-specific CBT, its specific and manual-driven nature has raised questions about its generalizability to real-life patients who often have comorbid disorders (Westen et al., 2004). Furthermore, the requirement for therapists to learn and implement distinct protocols for each disorder adds to their burden (Sauer-Zavala et al., 2017), potentially limiting the number of adequately trained CBT practitioners and, in turn, restricting patients’ access to treatment.

Transdiagnostic approaches to CBT have been developed to be applicable across multiple mental disorders by focusing on their commonality rather than surface-level symptoms (Mansell et al., 2009). Given the high comorbidity among depressive, anxiety, and related disorders (T. A. Brown et al., 2001; Hirschfeld, 2001; McGrath et al., 2020), transdiagnostic CBT offers a flexible treatment approach and may enhance the applicability of CBT. This approach can also reduce the time and financial costs for therapists to learn disorder-specific CBT (Craske, 2012; McEvoy et al., 2009). Furthermore, by addressing a broader range of psychopathology, transdiagnostic CBT may provide more comprehensive treatment for patients and lower treatment costs by reducing the time invested by therapists and patients (Schaeuffele et al., 2024).

The Unified Protocol for Transdiagnostic Treatment of Emotional Disorders (UP) is one approach to transdiagnostic CBT (Barlow et al., 2004, 2011). Emotional disorders are mental disorders characterized by frequent and intense experiences of negative emotions as well as an aversive reaction to these emotional experiences and attempts to escape or avoid them (Bullis et al., 2019). In the DSM-5, obsessive–compulsive disorder and post-traumatic stress disorder are excluded from anxiety disorders and classified under separate categories. However, the narrow definition of emotional disorders still includes anxiety disorders, which encompass obsessive–compulsive disorder, post-traumatic stress disorder, and related disorders, along with unipolar mood disorders. In a broader context, emotional disorders also encompass somatic disorders, borderline personality disorders, eating disorders, and insomnia (Bullis et al., 2019). Unlike disorder-specific CBT, which targets symptoms by using techniques derived from corresponding individual theoretical models, the UP focuses on neuroticism and emotional dysregulation, which are common factors underlying mechanisms of emotional disorders (Barlow et al., 2020; Wilamowska et al., 2010). The UP comprises eight modules: (1) Goal Setting and Motivation Maintenance, setting specific goals and learning the importance of maintaining motivation; (2) Psychoeducation, understanding the three components of emotions and the context before and after emotional experiences; (3) Mindful Awareness of Emotions, focusing on the non-judgmental recognition and acceptance of feelings; (4) Cognitive Flexibility, increasing the flexibility of thinking in situations that provoke emotions; (5) Countering Emotional Behaviors, replacing emotion-driven behaviors and emotion avoidance with more adaptive behaviors; (6) Enhancing Awareness and Tolerance of Emotion-Related Physical Sensations, increasing better understanding and acceptance of bodily sensations linked to emotions; (7) Emotion Exposures, confronting situations, stimuli, and physical sensations that provoke strong emotions; and (8) Relapse Prevention, planning to maintain progress and prevent regression (Barlow et al., 2011, 2017). The time spent on each module and the context within the module can be adjusted based on patient needs (Barlow et al., 2011, 2017).

Early studies on the effectiveness of the UP were initially conducted in an individual format (Boisseau et al., 2010; Ellard et al., 2010; Farchione et al., 2012) but later expanded to include a group format. A systematic review of transdiagnostic group CBT found that 11 of 32 studies of group CBT in adults used the UP, making it the most frequently implemented intervention (Harrison et al., 2016). In addition, a recent meta-analysis indicated that the group-format UP is effective in significantly reducing anxiety and depressive symptoms and improving quality of life and social adjustment (Ayuso-Bartol et al., 2024). Group therapy provides significant benefits in efficiency and cost-effectiveness for therapists by enabling the simultaneous treatment of more than one patient (J. S. Brown et al., 2011). In addition, the group format can facilitate normalization, reduce the stigma associated with treatment, and enhance motivation for treatment by having patients share their experiences with each other (Bullis et al., 2017). The advantages of group therapy are further enhanced by transdiagnostic approaches such as the UP, effectively addressing the challenge many clinics face in forming homogeneous patient groups (Erickson et al., 2007).

In studies of group-format UP, there is significant variation in the characteristics of participants as well as the intervention’s structure and components. Unlike the individual format of the first edition of the therapist’s guide (Barlow et al., 2011), which clearly outlines the treatment structure, there is no clear outline for group-format UP. Therefore, each study implements group-format UP differently in terms of treatment structure and module composition. For instance, module structure, the number of patients per therapist, the experience and training of therapists, the number of sessions, and implementation times are reported to vary based on local circumstances, culture, and region (Bullis et al., 2014; Corpas et al., 2022; Laposa et al., 2017; Osma et al., 2015; Reinholt et al., 2017, 2022). Various implementation innovations have also been reported, such as significantly increasing the time spent reflecting on homework within a session compared to individual sessions (Bullis et al., 2014) and using individual sessions in conjunction with interventions from the middle of the intervention (Powell et al., 2021). In some studies, the order of the modules in the group format UP was modified from the original individual format (Powell et al., 2021; Laposa et al., 2017). Moreover, although the UP is intended to address a broad range of emotional disorders, in some group-format UP studies, post-traumatic stress disorder was not included in the inclusion criteria (Powell et al., 2021; Reinholt et al., 2022), whereas in others, patients with post-traumatic stress disorder were specifically targeted (Varkovitzky et al., 2018). Hence, numerous aspects of treatment delivery, including the fields and settings where research is conducted, the diagnoses and characteristics of the target populations, the intervention structure and implementation strategies, and the experience and training of the providers, are critical for tailoring group-format UP to local contexts and providing suggestions for future research directions. However, to our knowledge, these aspects have not been comprehensively reviewed.

Although group-format UP shows promise as an effective method of delivering CBT, as supported by growing evidence worldwide, more organized and detailed information is needed. We have prepared a scoping review protocol to provide a comprehensive overview of the critical aspects that should be considered and adjusted to facilitate the implementation of group-format UP across diverse regions and contexts. Establishing such protocols helps prevent bias in the findings, enhances transparency, and ensures rigorous reporting (Peters et al., 2020). Accordingly, we developed this protocol in line with scoping review guidelines (Peters et al., 2020; Tricco et al., 2018) before conducting the review.

## 2. Materials and Methods

A scoping review is a method for comprehensively examining ongoing research by mapping a wide range of literature in order to identify gaps in the research (Peters et al., 2020). The proposed scoping review will be conducted in accordance with the Joanna Institute (JBI) (Peters et al., 2020) guidelines and the guidelines by Tricco et al. (2018) for reporting extensions to the PRISMA statement for scoping reviews (PRISMA-ScR). The outline of this protocol is divided into five stages: (1) identifying the research questions, (2) identifying relevant studies, (3) selecting the studies, (4) charting the data, and (5) collating, summarizing, and reporting the results, following the framework proposed by Arksey and O’Malley (2005).

### 2.1. Stage 1: Identifying Research Questions

The primary purpose of this research is to comprehensively map out the aspects necessary for adapting group-format UP for adults with symptoms related to emotional disorders. Although the UP in adolescents (UP-A) and the UP in children (UP-C) were developed from the UP for adults, these treatments were uniquely designed for these age groups. Therefore, it is expected that there will be few studies on the UP-A and UP-C in adults. However, if studies have implemented these interventions in adults aged 18 years and older, we will include them in this review. Moreover, other treatments have emerged, including modified versions of the UP and combinations of different psychotherapies. Given the difficulties in establishing a precise definition of what defines the UP (Cassiello-Robbins et al., 2020), we decided to include these variations within the scope of UP in this study. Based on this definition, we formulated four detailed research questions to accomplish the overarching objective.

1. In what fields and settings has group-format UP been utilized?
  This question explores the various areas where group-format UP has been implemented, including in the medical, educational, and industrial fields. We will also explore the specific characteristics of these settings, including research environments, hospitals, and academic institutions.
2. What was the target population?
  This question investigates the characteristics of the target population and the participants involved in the group-format UP. We will emphasize the presenting symptoms, including symptoms of primary diagnoses and comorbid emotional disorders. Additionally, we will gather information on the severity of these conditions, considering both the primary diagnosis and demographic factors such as average age and gender proportions among the participants.
3. Who delivered group-format UP?
  This question focuses on the characteristics of the therapists who implemented group-format UP. It examines their professions, licensure, training, and prior experience before implementing the program. Furthermore, we will collect information about the training and supervision provided to the therapists during the intervention periods.
4. How was group-format UP delivered, and what additional efforts were implemented beyond the manual?
  This question addresses the specifics of delivering group-format UP, including structural elements such as treatment length, session duration, frequency, and the overall duration of the intervention. It also examines the content of the intervention, noting aspects such as content compression, personal follow-up, and the inclusion of original material. Additionally, this question explores logistical details, including the number of participants per group, the presence of co-therapists, and the delivery methods (e.g., face-to-face or online).

### 2.2. Stage 2: Identifying Relevant Studies

Establishing a Population, Concept, and Context (PCC) framework is useful in scoping reviews to delineate the components integral to the studies.

- Population: This study’s target population is adults aged 18 years or older with symptoms of emotional disorders. The term *emotional disorders*, as defined by Barlow (Barlow et al., 2004), the founder of the UP, encompasses all anxiety and depressive disorders outlined in the DSM-IV. However, the most recent UP therapist guide broadens the scope to include mental disorders defined in the DSM-5, with adaptations extending to health-related anxiety, dissociation, and alcohol and substance abuse linked to intense negative emotions such as anxiety and depression (Barlow et al., 2017). Furthermore, Barlow and Farchione (2017) note the extensive application of the UP to various disorders and conditions. In this study, we will use a broader definition that includes participants exhibiting symptoms of emotional disorders. Additionally, to encompass group-format studies on the UP conducted in fields beyond the medical domain, our target population will consist of individuals with symptoms related to emotional disorders, both with and without a formal diagnosis.
- Concept: This study will attempt to answer research questions exploring the fields and settings where research on group-format UP is being conducted, the diagnoses and characteristics of the target populations, the experience and training of the providers, the implementation modalities of the inclusion of both face-to-face and non-face-to-face interventions, and the intervention structure and implementation strategies used in this format. The UP is acknowledged as an effective form of psychotherapy applicable to symptoms of a diverse range of disorders (Barlow & Farchione, 2017), and we anticipate that its implementation in a group setting will expand its potential impact.
- Context: Our inclusion criteria extend beyond the confines of the medical field, encompassing studies conducted in diverse settings, including schools and workplaces. It is also limited to studies publicly available in English.
- Study Type: This study will include articles on intervention studies, focusing primarily on randomized controlled trials with a control group and comparative studies that may consist of non-randomized and pre-post trials. Consequently, studies falling outside the purview of intervention studies, including qualitative and case studies, will be excluded.

#### Search Strategy

A three-step search strategy will be employed, following the JBI guidelines (Peters et al., 2020). The literature search will involve five online databases: PubMed, MEDLINE, Web of Science, PsycINFO, and ClinicalTrials.gov.

- Database Search: In October 2024, we conducted a thorough literature search to identify relevant studies, explicitly performing preliminary searches in four of the five online databases listed above. Through this process, we iteratively refined the search formula.
- Keyword Analysis: We then performed a keyword analysis that involved examining terms present in the titles and abstracts of the retrieved articles and identifying keywords specified within the articles. Subsequently, we conducted a second search using the identified keywords and search terms. This iterative process involved assistance from a librarian for guidance on refining the search formula.
- Citation Search: The next step is to search for additional sources of information based on the citations of the identified references. This phase will involve an examination of the citations of all identified sources, references to the text, citations in the UP, systematic reviews of group-format CBT, and so on. We will contact the authors of primary sources and literature reviews as needed. The search strategy common to all five databases is provided in the Appendix A.

### 2.3. Stage 3: Selection Process

The initial step will involve uploading the selected studies from all databases into Endnote. Subsequently, duplicates will be removed in the Endnote 21 software. A spreadsheet containing the studies’ titles, authors, and publication years will be generated to aid the screening process.

Throughout the selection process, four reviewers (M.K., N.K., M.N., and H.F.) will independently assess the titles and abstracts, making decisions based on predetermined inclusion and exclusion criteria. The exclusion reasons will be documented. The reviewers will meet at the abstract-review phase’s beginning, middle, and end to address any issues related to the study selection. Discrepancies between reviewers will be resolved through discussions involving a fifth reviewer (M.I.) to achieve consensus.

The full text will be displayed when additional information is required, and a decision will be made after reviewing the contents of the article. Efforts will be made to contact the author(s) to obtain the required information before making a decision. However, the data will be excluded if no response is received in a reasonable period of time and the requested data cannot be obtained. A flowchart for the literature selection process will be created, following the guidelines set by the PRISMA-ScR. We expect to complete these full-text screenings in February 2025. The inclusion and exclusion criteria will be established accordingly.

#### 2.3.1. Inclusion Criteria

For a study to be included in this scoping review, it must satisfy the following criteria: (1) intervention employing the UP, (2) implementation of group therapy in the intervention, (3) the outcome includes scales for measuring symptoms related to emotional disorder, including depression, anxiety, and neuroticism, (4) the participants are aged 18 years or older, and (5) studies that are publicly available in English.

#### 2.3.2. Exclusion Criteria

Non-intervention studies will be excluded from this scoping review.

### 2.4. Stage 4: Data Charting

A spreadsheet will record the selected studies during the full-text screening (Stage 3), and the four independent reviewers (M.K., N.K., M.N., and H.F.) will populate the table. An initial version of the table is provided in the Appendix B. The spreadsheet can be revised to accommodate additional data that might enhance the data-extraction process. Before data extraction, the research team will discuss the process and data items in response to such adjustments.

In the case of missing data or when additional data are required, we will contact the authors to request the necessary information to facilitate the decision-making process. The two reviewers will discuss any discrepancies during the data extraction process. Any disagreements will be resolved through discussions with a fifth reviewer (M.I.) as necessary.

A qualitative-descriptive process will precede a quantitative evaluation based on indicators such as number and frequency for all outcomes. Charting will be performed as needed, and preliminary charts are included in the Appendix B. We expect to complete the data-extraction processes by May 2025.

### 2.5. Stage 5: Collating, Summarizing, and Results Reporting

This stage comprises the following three key phases, following the guidelines of Levac et al. (2010).

1. Analyzing the data.
2. Quantitative and qualitative analyses will be performed using charts created during the data charting stage to address the research question. The quantitative analysis will include total data (an overview of all studies), frequency (the number of studies for each study design, the number of studies in each country, the number of studies for each diagnosis or condition, and the number of studies per intervention type), and averages or ranges (the age of participants, session time, number of sessions, the number of therapists in the intervention, and the dropout rate). The qualitative analysis will identify trends in intervention structure, content, type, therapist characteristics, intervention fidelity, and outcomes of the studies by target diagnosis or condition and its severity, field/setting of practice, and country/region of implementation. Furthermore, all studies will undergo a qualitative evaluation using the corresponding JBI critical appraisal tools in their study designs (e.g., randomized controlled trials (Barker et al., 2023) and quasi-experimental studies (Barker et al., 2024).
3. Reporting the results.
4. Based on the mapped data, a clear answer to the research question will be provided. The results, such as text, figures, tables, and graphs, will be presented appropriately.
5. Applying meaning to the results.
6. The implications of the overall research objectives and considerations for future research, practice, and policy will also be discussed.

We expect to complete these analyses by November 2025.

## 3. Discussion

This will be the first scoping review to summarize the critical aspects associated with a group format of the UP intervention for symptoms related to emotional disorders in adults. The review will map a diverse array of information to comprehensively elucidate how the UP is effectively applied in a group setting. The findings from this review are expected to provide valuable insights into adapting and implementing group-format UP within local circumstances, policies, and cultural contexts across different regions of the world, particularly in areas with limited resources. Additionally, this review will identify key aspects that warrant further exploration, including the field of application, target population, delivery methods, and therapist training associated with group-format UP. These findings will provide recommendations and implications to enhance the clinical application and research development of group-format UP, thereby contributing to the dissemination of evidence-based psychological interventions.

## Data Availability

No new data were created or analyzed in this study. Data sharing is not applicable to this article.

## Appendix A. Search Formula and Preliminary Search in Databases

Search formula: “Group” AND “Unified Protocol*”.

## Appendix B. Categories of Data Extraction Forms

**Key Information**	**Research Question**	**Domain**	**Extract Data Items**
Author(s)	-	Author(s)	- Author(s)
Year of publication	-	Year of publication	- Year of publication
Country of origin	-	Country of origin	- Country of origin
Fields and settings	In what fields and settings has group-format UP been utilized?	Implemented fields	- Fields
		Characteristics of the settings	- Settings
Population and sample size	Who was the target population?	Target population and participants’ characteristics	- Disorders encompass the primary diagnosis, any comorbid conditions, as well as presentations that are subthreshold or otherwise characterized - Severity based on the primary diagnosis - Mean age - Gender ratio
	-	Sample-size	- Sample size
Methodology/methods	-	Study design	- Study design
Intervention characteristics	How was group-format UP delivered, and what additional efforts, beyond following the manual, were implemented?	Intervention type	- Delivery methods: face-to-face/online, etc.
		Duration	- Duration - Frequency - Number of sessions - Duration per session
		Number of therapists and participants per group	- Mean number of participants per group - Mean number of therapists and co-therapists present per group
		Content	- Intervention content: content compression, personal follow-up, original content, etc.
Therapist characteristics	Who delivered group-format UP?	Therapist profession and licensure	- Therapist profession and licensure
		Therapists’ experience and training	- Experience before delivering group-format UP - Training before delivering group-format UP - Qualifications and certifications related to the UP
		Therapist training and supervision while delivering group-format UP	- Training and supervision during the delivery of group-format UP
		Fidelity	- Fidelity
Outcome measures	-	Outcome	- Measurements of symptoms related to emotional disorders - Primary outcome and secondary outcome
Findings	-	Findings	- Difference in effect at pre-post and follow-up - Diagnosis after the treatment - Dropout rate
